# Organic composition of starch wastewater steers denitrifying microbiomes for enhanced nitrogen removal

**DOI:** 10.1016/j.isci.2026.116217

**Published:** 2026-06-02

**Authors:** Xiaoya Guo, Yu Liang, Haihong Yan, Yuegang Nian, Fengyun Bu

**Affiliations:** 1 State Key Laboratory of Environmental Criteria and Risk Assessment, Chinese Research Academy of Environmental Sciences, Beijing 100012, P.R. China; 2 Research Center of Environmental Pollution Control Engineering Technology, Chinese Research Academy of Environmental Sciences, Beijing 100012, P.R. China

**Keywords:** environmental monitoring, water resources engineering, environmental engineering

## Abstract

Two-stage corn starch wastewater, specifically hydrolysis acidification wastewater (HAW) and vertical flow sedimentation wastewater (VSW), was assessed as an external carbon supplement to enhance denitrification in low C/N municipal wastewater. In the initial rapid phase, NOx-N reduction rates reached 15.91 and 18.04 mg N g^−1^ MLVSS h^−1^ for HAW and VSW, respectively, slowing significantly to 3.57 and 8.02 mg N g^−1^ MLVSS h^−1^ during the subsequent nitrite reduction phase. EEM-PARAFAC indicated preferential depletion of a tyrosine-like component linked to fast NOx removal. *Nir*-gene profiling showed *nirK* denitrifiers were more carbon-sensitive than *nirS*, and *Rhizobium* dominated *nirK* under VSW addition. Thirty-day operation remained stable with low effluent nitrate and limited nitrite accumulation. This work supports the feasibility of recycling corn starch process wastewaters, particularly VSW, as external carbon sources for nitrogen removal and proposes an engineering pathway for implementation.

## Highlights

• Corn starch wastewater serves as an effective supplementary carbon source
• High *NirK-denitrifier* abundance is critical for rapid nitrite reduction
• VSW selectively enriches *Rhizobium* to dominate *NirK*-mediated denitrification
• Real-time pH trajectories act as diagnostic markers for process control

## Graphical abstract

**Figure:**
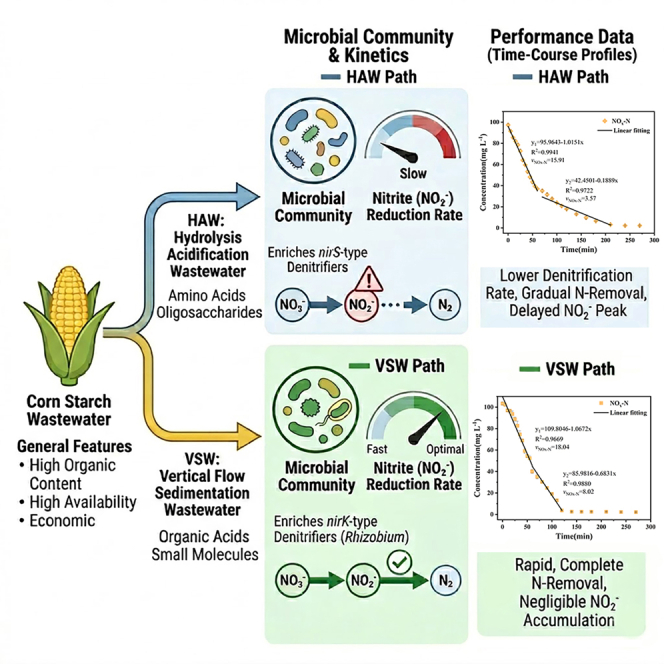

## Introduction

Starch is a fundamental carbohydrate and an important source of functional ingredients in both food and non-food industries.^1^ Globally, corn currently supplies over 80% of the world’s starch production.^2^^,^^3^^,^^4^ However, substantial quantities of corn starch wastewater (CSW) are generated during production, presenting significant environmental challenges. In China alone, the annual output of CSW exceeds 1,135 million tons, with chemical oxygen demand (COD) ranging from 2,000 to 29,300 mg L^−1^ and containing sugars, proteins, vitamins, and inorganic salts.^5^^,^^6^^,^^7^ To overcome the high energy demand, costly operations, and limited treatment efficiency of physicochemical methods, researchers have explored various resource recovery and reuse pathways, such as hydrogen production, lipid accumulation, and microbial fuel cells.^8^^,^^9^^,^^10^^,^^11^ However, large-scale industrial applications remain limited by complicated pretreatment procedures and high operational costs.

Stringent total nitrogen (TN) discharge regulations have driven WWTPs to seek novel strategies for enhanced nitrogen removal. Many WWTPs encounter influents with low carbon-to-nitrogen (C/N) ratios (<4.0), resulting in limited denitrification.^12^ Although small amounts of ammonia, nitrite, and organic nitrogen may be present, nitrate is typically the predominant nitrogen form in the final effluent. Hence, improving nitrate removal in biological treatment steps is pivotal for meeting stringent TN discharge limits.^13^ Biological denitrification is generally recognized as the most cost-effective approach, wherein denitrifying bacteria convert nitrate to nitrogen gas under oxygen-limited conditions, using organic matter as the terminal electron donor.^14^^,^^15^ When external carbon sources are introduced, particularly those derived from waste streams, they can significantly enhance the denitrification process and foster the principles of a circular economy.^16^

To enhance biological denitrification, studies have focused on adding external organic carbon sources, including liquid-phase organics (acetate, industrial wastewaters, fermentation by-products),^17^^,^^18^^,^^19^^,^^20^ solid carbon sources,^21^^,^^22^ and gaseous substrates.^23^^,^^24^ Among these, small-molecule organics remain dominant in municipal WWTPs due to high bioavailability. However, their high costs, secondary pollution risks, and excessive sludge production have spurred interest in sustainable alternatives.^25^ Natural cellulose-based solid carbon sources offer dual functionality as carbon releasers and biofilm carriers. Yet, their performance is highly sensitive to environmental conditions and may still pose secondary pollution risks. Biodegradable polymers (BDPs) also face limitations due to high production costs, despite recent efforts to develop composite materials combining agricultural residues with BDPs. While promising, such hybrid systems require further validation for practical applications.

Emerging studies highlight waste-derived liquid carbon sources, such as industrial effluents, sludge hydrolysates, and food-waste streams, which align with circular economy principles by converting waste into resources. Thermally hydrolyzed sludge filtrate increased nitrogen removal in A^2^O systems from 55.3% to 74.3%,^26^ while fresh plant biomass in constructed wetlands improved removal rates from 18.6% to 51.6%.^27^ Food-waste effluents can supply organic substrates for denitrifiers.^28^^,^^29^ However, inconsistent degradation kinetics may lead to incomplete denitrification and nitrite accumulation (0.95-‌37.8 mg N g^−1^ MLVSS h^−1^).^30^^,^^31^ Two critical knowledge gaps persist: first, a mechanistic understanding of how carbon source structural complexity governs electron donor bioavailability, metabolic pathways, and rate-limiting steps. Second, the functional dynamics of *nirK*- and *nirS*-type denitrifiers under varying carbon regimes, which currently hinder targeted microbial regulation for enhanced performance.

CSW represents a viable alternative carbon source, characterized by high availability, low cost, and rich biodegradable compounds (e.g., sugars, VFAs, proteins).^24^ However, its compositon varies markedly across starch processing stages, posing challenges for denitrification optimization. How do the structural complexity and composition of starch-based waste carbon sources regulate denitrification kinetics, microbial community assembly, and nitrogen removal efficiency by modulating electron donor bioavailability, metabolic pathways, and functional guild activities?

Here, we systematically evaluate vertical-flow sedimentation wastewater (VSW) and hydrolysis-acidification wastewater (HAW) from a corn starch plant to: (1) compare their denitrification performance and nitrite accumulation patterns against conventional synthetic carbon sources; (2) establish correlations between organic matter characteristics and denitrification kinetics; (3) characterize the dynamic responses and ecological succession of *nirK*- and *nirS*-type denitrifiers under different carbon-source regimes. This work provides actionable insights for implementing CSW in nitrogen removal systems, advancing circular bioeconomy principles while replacing synthetic carbon sources.

## Results and discussion

### Nitrogen removal performance

**Figure 1:**
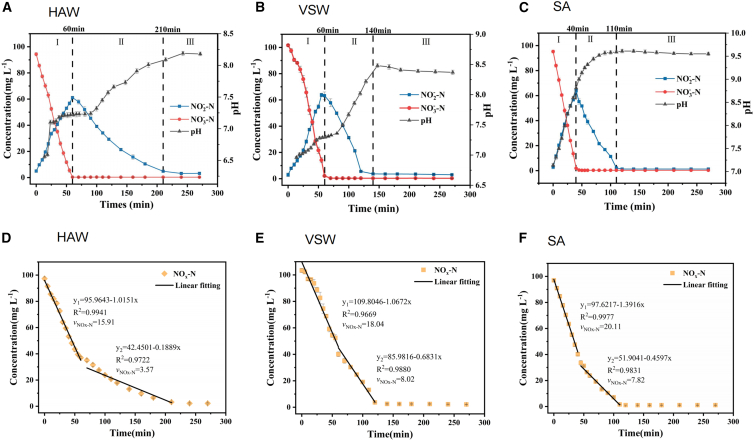
Denitrification performance and variation of pH with different carbon sources Time-course profiles of NO_3_–N, NO_2_–N, and pH for HAW (A), VSW (B), and SA (C); linear fitting of NO_x_-N removal for HAW (D), VSW (E), and SA (F). Data are represented as mean ± SD.

Nitrogen dynamics obtained with VSW and HAW amended reactors were benchmarked against a sodium-acetate (SA) control. Time-course profiles of NO_3_^−^-N, NO_2_^−^-N, and pH are shown in Figures 1A–1C. Following Zhang et al.,^32^ the denitrification process was divided into three stages: stage I, rapid NO_3_^−^ reduction; stage II, NO_2_^−^ reduction; stage III, post-denitrification/fermentation. In stage I (0–60 min for VSW and HAW; 0–40 min for SA), the concentration of NO_3_^−^-N decreased rapidly, while NO_2_^−^-N accumulated. NO_3_^−^-N removal rates were 99.7% for HAW, 97.9% for VSW, and 98.0% for SA. Peak NO_2_^−^-N concentrations were 61.2 mg L^−1^ for HAW, 63.2 mg L^−1^ for VSW, and 59.1 mg L^−1^ for SA. Linear fitting of NO_x_-N removal (Figures 1D–1F) yielded stage-I specific rates of 15.91 mg N g^−1^ MLVSS h^−1^ (HAW), 18.04 mg N g^−1^ MLVSS h^−1^ (VSW), and 20.11 mg N g^−1^ MLVSS h^−1^ (SA). The faster SA rate reflects the higher bioavailability of acetate. In Stage II (60–210 min for HAW; 60–140 min for VSW; 40–110 min for SA), once nitrate was exhausted, NO_2_^−^-N declined steadily. Stage-II lasted 150 min with HAW but only 80 min with VSW and 70 min with SA, illustrating more efficient nitrite reduction in the latter two systems. Corresponding rates were 3.57 mg N g^−1^ MLVSS h^−1^ (HAW), 8.02 mg N g^−1^ MLVSS h^−1^ (VSW), and 7.82 mg N g^−1^ MLVSS h^−1^ (SA). The approximately 2.25- to 4.46-fold decrease from stage I confirms that nitrite reduction is the rate-limiting step. The slower kinetics with HAW may stem from its higher fraction of complex macromolecules that release electrons more slowly to nitrite reductase. The reduction rate of nitrite was significantly lower than that of nitrate, possibly due to the considerable difference in electron flow for nitrate and nitrite reductase. Nitrate is considered to be the preferred electron acceptor,^33^ resulting in nitrite accumulation. The reason for the difference in nitrite reduction rates between HAW and VSW may be related to the substrate metabolism of denitrifying bacteria. In stage III (>210 min for HAW, >140 min for VSW, >110 min for SA), after nitrite removal, nitrogen species stabilized. Residual organics underwent slow anaerobic fermentation, reflected by minor pH falls at the end of the cycle.

**Figure 2:**
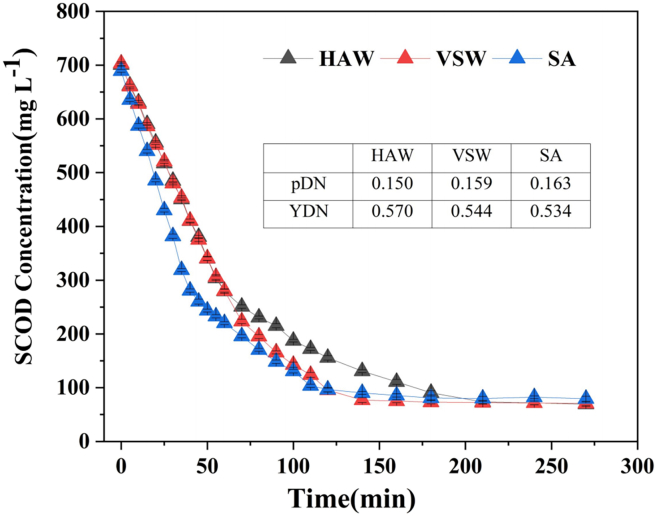
The degradation of SCOD during denitrification with different carbon sources Data are represented as mean ± SD

The degradation of soluble chemical oxygen demand (SCOD) further reflects the utilization of the external carbon sources during denitrification (as shown in Figure 2). SCOD was consumed rapidly during stage I, and then approached a plateau after stage II. SCOD utilization rates at the end of stage II were 89.50%, 89.04%, and 84.97% for HAW, VSW, and SA, respectively. The corresponding potential of denitrification (P_DN_) were 0.150, 0.159, and 0.163 mg NO_x_-N mg^−1^ SCOD. As the external carbon source for denitrification, VSW possessed the closest value to commercial SA, indicating that the VSW had the highest potential to serve as a promising carbon source for denitrification. In addition, the yield of denitrification (Y_DN_) of SA was the lowest at 0.534 gSCOD/gSCOD, followed by VSW (0.544 gSCOD/gSCOD), and HAW (0.570 gSCOD/gSCOD). A low Y_DN_ during denitrification indicates that, upon carbon dosing, denitrifying activated sludge allocates a larger fraction of the consumed carbon to NOx reduction rather than to biomass synthesis, thereby sustaining nitrogen removal while mitigating excess sludge production and the associated disposal costs.^34^

The variation of pH with different carbon sources is also depicted in Figure 1. During denitrification with organic electron donors, alkalinity is generated due to the production of bicarbonate and carbonate ions.^35^ As illustrated in Figure 1, a consistent increase in pH was observed during denitrification. When CSW were used as external carbon sources, the buffering capacity was greater in both VSW (ΔpH 1.4) and HAW (ΔpH 1.6) systems compared to the sodium acetate control (ΔpH 2.4). Thus, carbon-source type strongly affects pH dynamics during treatment. The reduced pH fluctuation in starch wastewater likely stems from intermediate acidification steps and CO_2_ accumulation during the metabolism of complex organics, such as proteins and polysaccharides, which partially neutralize alkalinity. For instance, hydrolysis of starch-derived macromolecules releases low-molecular-weight organic acids and amino acids, counteracting the alkalinity generated during denitrification.

The stoichiometric analysis further elucidates these differences. Denitrification using SA as the electron donor (Equation 1) produces higher quantities of carbonate and bicarbonate, driving a pronounced pH rise:

(Equation 1) $$0.99{CH}_{3}{COO}^{-}+{NO}_{3^{-}}\to {0.12C}_{5}H_{7}O_{2}N+{0.44N}_{2}+{0.64CO}_{3^{2-}}+{0.69H}_{2}O+{0.71HCO}_{3^{-}}$$

In contrast, starch wastewater metabolism (Equations 2) yields CO_2_ as a by-product, moderating alkalinity accumulation:

(Equation 2) $${5C}_{6}H_{10}O_{5}+{24NO}_{3^{-}}\to {12N}_{2}+{24HCO}_{3^{-}}+{6CO}_{2}+{13H}_{2}O$$

Note that C_6_H_10_O_5_ represents a simplified formula for starch; CSW also contains lipids, proteins, and other organics. Therefore, a more detailed stoichiometric model is required.

The pH trajectory (Figure 1) reveals three distinct denitrification stages. In stage I (nitrate reduction), the pH slope is lower than in stage II (nitrite reduction), attributed to divergent electron acceptor utilization and by-product profiles. Specifically, nitrate reduction generates CO_2_, whereas nitrite reduction produces both CO_2_ and OH^−^, amplifying alkalinity in stage II. During stage III, nitrate/nitrite depletion triggers anaerobic fermentation of residual organics, releasing acids (e.g., VFAs) that reverse prior alkalinity gains.

Additionally, buffering capacity also critically modulates pH dynamics.^36^ Even if equivalent amounts of alkalinity are theoretically produced, the presence of various acid-base pairs can moderate or mask pH changes. In the case of HAW, hydrolytic enzymes and acidogens convert macromolecules to transient VFAs, inducing localized acidification before denitrifiers consume these acids. This interplay between acidogenesis and alkalization explains the persistently lower pH in HAW compared to SA systems.

### Organic matter removal performance

**Figure 3:**
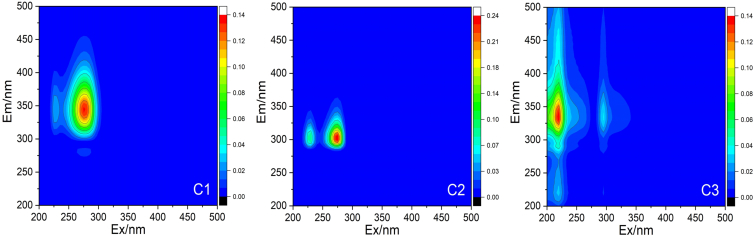
Three fluorescence components identified by the PARAFAC model of HAW and VSW systems

To elucidate the dynamics of organic matter utilization during denitrification, fluorescence components in wastewater carbon sources were characterized through EEMs-PARAFAC analysis. This approach aimed to resolve substrate-specific variations in electron donor utilization and their correlation with denitrification kinetics. Three distinct fluorescent components were identified in the HAW system, labeled as C1 (225/275 nm, 350 nm), C2 (230/275 nm, 305 nm), and C3 (220/275 nm, 335 nm), as shown in Figure 3. The excitation and emission loading were plotted in Figure S2. The VSW system only showed the presence of C1 and C2. Based on previous literature and the fluorescence peak types, C1 was identified as tryptophan-like substances, C2 as tyrosine-like substances, and C3 as indicative of soluble microbial metabolites.^37^^,^^38^

**Figure 4:**
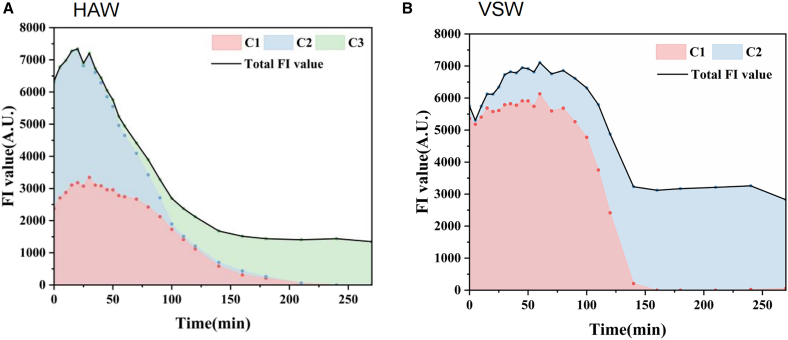
Changes in fluorescence intensity of DOM in the systems of HAW and VSW

Fluorescence intensity varied heterogeneously during denitrification processes, attributable to the differential composition of dissolved organic matter (DOM). In the HAW system, stage І (0–60 min) exhibited rapid depletion of component C2, fluorescence intensity declined from 3945 to 1905 A U., with a reduction of 51.7% (Figure 4A). During this period, the NO_x_-N removal rate reached 15.91 mg N g^−1^ MLVSS h^−1^. Pearson correlation analysis revealed a strong linear correlation between C2 degradation and nitrogen removal, with correlation coefficients of 0.90 (*p* < 0.01) for NO_3_^−^-N and 0.91 (*p* < 0.01) for NO_x_-N. This underscores C2 as the dominant electron donor during this phase. Stage II (60–210 min) displayed a substrate utilization shift, where C1 became the primary substrate. Despite high correlation between C1 consumption and nitrogen removal (*r* = 0.99, *p* < 0.01) (Table S2), its slower degradation kinetics reduced NO_x_-N removal efficiency to 3.57 mg N g^−1^ MLVSS h^−1^. Concurrently, C2’s correlation with denitrification decreased significantly (*r* = 0.83, *p* < 0.01), indicating reduced functional dominance. Notably, component C3 exhibited progressive accumulation, with fluorescence intensity increased from 23 to 1,349 A U., demonstrating strong inverse correlations with nitrogen reduction across both stages (*r* = −0.91, *p* < 0.01 in stage I; *r* = −0.99, *p* < 0.01 in stage II). This pattern suggests C3 represents soluble microbial byproducts generated during heterotrophic metabolism, potentially acting as electron shuttles while accumulating due to incomplete mineralization.

Contrary to the HAW system, VSW exhibited unique fluorescent signatures during denitrification (Figure 4B). Stage I (0–60 min) displayed a 23.68% increase in total fluorescence intensity from 5,747 to 7,108 A U., indicating limited microbial utilization of fluorescent DOM components during nitrate-to-nitrite reduction. Pearson correlation analysis (Table S3) revealed significant negative correlations (r = −0.86 to −0.89, *p* < 0.01) between the fluorescence intensity of components C1/C2 and nitrate concentrations, with concomitant accumulation rates of 11.4 and 12.9 A U. min^−1^, suggesting substrate preservation under partial denitrification conditions. In stage II (60–140 min), C2 decreased markedly from 6132 A U. to 2416 A U., while C1 accumulated to 2461 A U. This phase specific pattern demonstrates preferential utilization of low-molecular-weight tyrosine derivatives (C2) during nitrite reduction, achieving denitrification rates comparable to sodium acetate controls. Notably, C2 degradation fluorescence showed strong negative correlations with NO_2_^−^-N and NO_x_-N (r = −0.95, *p* < 0.01), whereas C1 showed a negative positive correlation with NO_2_^−^-N and NO_x_-N (r = 0.91, *p* < 0.01), suggesting potential redox-mediated transformation or electron-shuttling behavior.

The distinct substrate utilization patterns between HAW and VSW systems emphasize the critical role of carbon source composition in denitrification kinetics. Rapid degradation of tyrosine-like substances (C2) drives high initial rates, whereas complex substrates like tryptophan derivatives (C1) limit long-term efficiency. These findings align with microbial metabolic preferences for labile monomers.

### Biodiversity analysis and comparison

In order to gain a comprehensive understanding of the variation in microbial community structure across different denitrification assays and to elucidate the internal relationship between the structure of the microbial community and the denitrification reaction, samples were collected from the systems fed with VSW and HAW as carbon sources and then analyzed by sequencing. The system of no addition carbon source (NA) was operated as the control experiment.

Good’s coverage values across all samples were above 0.99 (see Table 1), indicating that the sequences obtained sufficiently cover the major bacterial phylotypes. The Chao index increased from 604 (NA) to 631 (VSW) and 608 (HAW), suggesting that the microbial richness in the denitrification system with VSW is greater than that of HAW. This difference may be attributed to VSW containing a variety of organic carbon-bearing components, which provided diverse metabolic substrates for microbial proliferation.^39^ Shannon and Simpson indices revealed significantly higher microbial diversity in carbon-supplemented systems (VSW: Shannon = 5.30, Simpson = 0.93; HAW: Shannon = 5.31, Simpson = 0.93) compared to NA (Shannon = 4.90, Simpson = 0.89), underscoring the role of carbon availability in enhancing microbial diversity. However, negligible differences between VSW and HAW suggest that carbon source type exerts limited influence on diversity, whereas its presence is the critical driver.

**Table 1:** Richness and diversity estimators of the bacterial sequences with different carbon sources

Sample	Good’s coverage	OTU	ACE	Chao	Shannon	Simpson
VSW	99.92%	606	642	631	5.30	0.93
HAW	99.95%	597	618	608	5.31	0.93
NA	99.94%	587	613	604	4.90	0.89

### Microbial community structure

**Figure 5:**
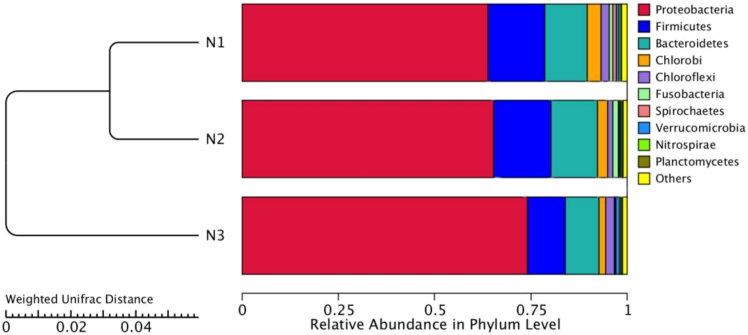
Variations of the microbial community structure of the samples at phylum (N1: VSW; N2: HAW; N3: NA)

Figure 5 illustrates the relative abundance of microorganisms under the three carbon-source regimes. The distribution of microorganisms was predominantly observed within the *Proteobacteria* phylum, accounting for 63.90%, 65.29%, and 61.20% of the total reads in VSW, HAW, and NA, respectively. The phylum *Proteobacteria* contains the majority of bacteria involved in nitrogen fixation, denitrification, and phosphate removal, which is the most dominant phylum of denitrifying bacteria.^40^ The systems contained a high abundance of *Firmicutes*, *Bacteroidetes*, *Chlorobi*, and *Chloroflexi*, and their respective abundances were 14.76%, 10.95%, 3.56%, 2.12% for VSW, and 14.86%, 12.09%, 2.71%, 1.31% for HAW. Firmicutes participate in denitrification processes and hydrolyze macromolecules such as cellulose, starch, and proteins.^41^ The relative abundance of the phylum *Proteobacteria* decreased in the CSW system compared to the NA, while the relative abundance of *Firmicutes* showed the opposite trend. This might be seen as the microbial populations adapting to the newly available carbon supply. External carbon enriched specific taxa and reduced minor populations.^42^ The co-dominance of *Proteobacteria* and *Firmicutes* implies a metabolic division of labor. In each system, the phylum *Nitrospirae* displayed abundances of 0.51%, 0.33%, and 0.38% for VSW, HAW, and NA, which was expected to play a crucial role in nitrite oxidation. This indicated that *Nitrospirae* is more adaptable to a variety of additional carbon sources and selection pressures.

### Versatility of denitrifying bacteria

The reduction of nitrite to nitric oxide (NO) is the defining and rate-limiting step of denitrification, distinguishing it from other dissimilatory nitrate reduction pathways. This process is primarily governed by nitrite reductases encoded by the *nirS* and *nirK* genes. Due to the high phylogenetic diversity of denitrifying bacteria, 16S rRNA gene-based analyses are often inadequate for their identification. Therefore, targeting the functional marker genes *nirS* and *nirK* provides a more accurate characterization of denitrifying communities.^43^

**Figure 6:**
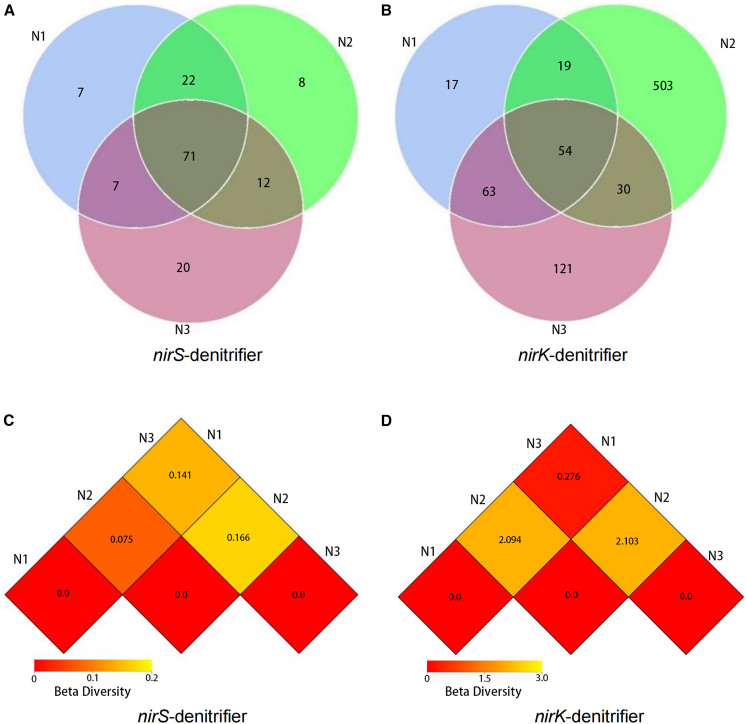
Similarity analysis of nirS- and nirK-type denitrifiers in different systems (N1: VSW; N2: HAW; N3: NA) Venn diagram illustrating community overlap (A and B), and beta-diversity based on weighted UniFrac distance (C and D).

The overlap of denitrifying communities across the three systems is shown in Figures 6A and 6B. A subset of species was common to all samples, with shared operational taxonomic units (OTUs) accounting for 47.65% and 6.67% of the *nirS* and *nirK* gene pools, respectively. This indicates that variations in CSW composition exerted a more pronounced effect on the community structure of *nirK*-type denitrifiers compared to *nirS*-type denitrifiers. Significant differences were observed among *nirK*-type denitrifiers in all sludge samples, particularly within the system supplemented with HAW as a carbon source. This trend was corroborated by beta diversity analysis based on Weighted UniFrac distance (Figures 6C and 6D). The results revealed substantially greater divergence between the communities fed VSW and HAW for *nirK-*type denitrifiers (beta diversity value of 2.094) than in *nirS-*type denitrifiers (value of 0.075), with the HAW system exhibiting the most distinct microbial profile.

**Figure 7:**
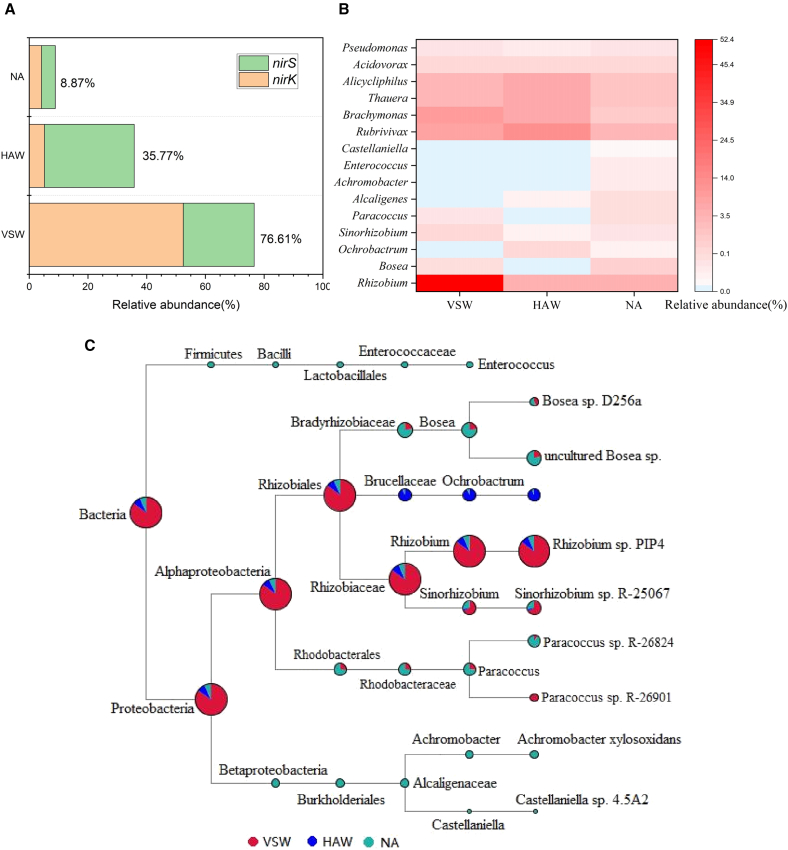
Composition characteristics of nirS- and nirK-type denitrifiers in different systems Relative abundance of denitrifying bacteria (A); Species classification tree of nirK-type denitrifiers (B); Heatmap of relative abundance of dominant denitrifying bacterial genera (C).

Changes in the abundance and composition of *nirS* and *nirK*-type denitrifiers are presented in Figure 7A. The addition of VSW and HAW as external carbon sources significantly stimulated the growth of both denitrifier types. The total relative abundance increased dramatically to 35.77% in HAW and 76.61% in VSW systems, compared to an initial abundance of 8.87% in the NA system, highlighting the role of bioavailable carbon in enhancing denitrifier biomass and metabolic activity.^44^ Notably, the growth potential of *nirK*-type denitrifiers was highest in the VSW system, where their biomass was approximately 10-fold greater than in the HAW system. This elevated biomass correlated with a higher nitrite denitrification rate, comparable to systems using SA as a carbon source. Enhanced microbial activity facilitated effective nitrate removal, consistent with prior findings.^45^

At the genus level, distinct successional patterns were observed (Figure 7B). *Rhizobium*, a dominant *nirK*-denitrifier known for its nitrogen-fixing capabilities in both symbiotic and free-living phases,^46^ showed markedly different abundance between systems with values of 5.1% in HAW and 52.4% in VSW. Its increased presence following CSW amendment suggests a potential key role in nitrite elimination. Conversely, the relative abundance of the aerobic denitrifier *Bosea* and the chemolithoautotrophic bacterium *Paracoccus* decreased from 0.11% (NA) to 0.03% (VSW) and 0.001% (HAW), and from 0.05% (NA) to 0.02% (VSW) and 0.001% (HAW), respectively. This decrease may reflect the susceptibility of these microorganisms to changes in ecological niches.^47^

Phylogenetic analysis of *nirK*-type denitrifiers further elucidated these community shifts (Figure 7C). The tree revealed clear clustering patterns corresponding to distinct taxonomic groups. Notably, sequences derived from the VSW system formed a well-supported and highly diverse clade, indicating selective enrichment of specific *nirK*-harboring lineages in response to its complex carbon composition. In contrast, sequences from the HAW system exhibited limited diversification and were frequently interspersed among other groups, suggesting a more conserved denitrifying community under these conditions. The stark contrast in *nirK*-denitrifier biomass between VSW and HAW underscores the role of carbon composition in shaping functional guilds. The heterogeneous carbon profile of VSW likely fostered a synergistic microbial network. For instance, Firmicutes may initially hydrolyze starch into oligosaccharides, which are subsequently utilized by *Rhizobium* for denitrification. This cross-feeding synergy minimizes intermediate accumulation and enhances overall nitrate removal efficiency. Conversely, the linear amylose structure in HAW may limit such metabolic interactions, promoting competition among taxa like *Thauera* and *Rubrivivax* for substrates, leading to incomplete denitrification and nitrite accumulation. It is worth noting that the persistence of *Nitrospirae* (0.33%–0.51%) across all systems highlights functional redundancy in nitrite oxidation, ensuring system stability under varying carbon loads. Similarly, the overlap observed among *nirS* taxa suggests metabolic backup capacities that buffer the community against functional collapse during carbon source transitions.

The *nirS*-type denitrifying community was dominated by *Rubrivivax* (8.28% in VSW, 13.30% in HAW), *Brachymonas* (9.59% in VSW, 5.33% in HAW), *Thauera* (2.83% in VSW, 6.57% in HAW), and *Alicycliphilus* (3.33% in VSW, 5.33% in HAW). *Rubrivivax* is capable of degrading aromatic hydrocarbons via phototrophic and chemotrophic pathways.^48^ *Brachymonas* is a common denitrifier in wastewater systems, utilizing cyclic alkane carbon for growth.^49^ *Thauera* can denitrify a range of carbon sources, including aromatic molecules.^50^ *Alicycliphilus* exhibits metabolic versatility, biodegrading xenobiotics under both oxic and anoxic conditions by utilizing oxygen, nitrate, or chlorate as terminal electron acceptors.^51^ The study suggests that *nirS*-type denitrifiers were more resilient to external disturbances compared to *nirK*-type denitrifiers. It was worth mentioning that the abundance of *Thauera* in the HAW system was higher than that of VSW. The bacterium has been reported as denitrifier with the *narG* and *narI*/*narV* genes, leading to high nitrite accumulation.^52^ This potentially explains the lower nitrite reduction rate observed in HAW.

### Environmental implications

This section evaluates the operational feasibility of reusing stage-specific corn-starch wastewaters (HAW/VSW) as external carbon sources in sequencing batch reactors (SBRs) and summarizes practical implications for carbon-source selection, monitoring, and process control.

**Figure 8:**
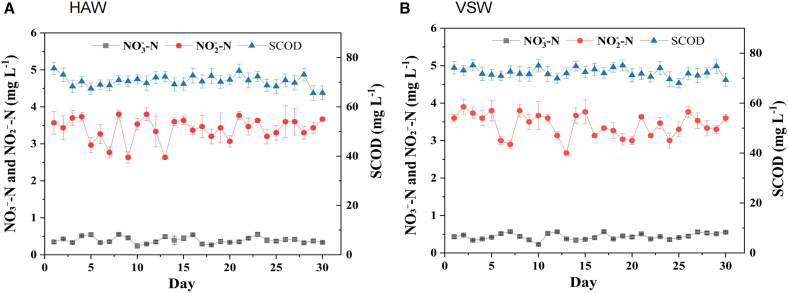
Long-term stability during 30-day SBR operation with different carbon sources Data are represented as mean ± SD.

To assess operational stability, SBRs fed with VSW or HAW were monitored for 30 consecutive days by measuring effluent NO_3_^−^-N, NO_2_^−^-N, and SCOD at the end of the decanting phase. Over the monitoring period, effluent NO_3_^−^-N remained consistently low for both carbon sources (HAW: 0.441 ± 0.087 mg/L; VSW: 0.394 ± 0.093 mg L^−1^). Effluent NO_2_^−^-N was also stable (HAW: 3.406 ± 0.349 mg L^−1^; VSW: 3.397 ± 0.369 mg L^−1^), with all VSW observations below 4.0 mg L^−1^, whereas HAW showed occasional values up to 4.1 mg L^−1^. Meanwhile, effluent SCOD remained relatively stable throughout the 30-day operation, with average concentrations of 70.46 ± 0.39 mg/L for HAW and 72.25 ± 2.70 mg/L for VSW, indicating consistent residual organic levels and steady carbon utilization under both carbon-source regimes. Together, these results indicate sustained low residual nitrate, limited nitrite accumulation, and stable organic matter profiles during long-term operation (Figures 8A and 8B). To explain this stable performance and the subtle differences in nitrite accumulation between VSW and HAW, the discussion in further section links effluent profiles with carbon-source composition, microbial metabolic interactions, and functional denitrifier communities.

The integration of carbon source characteristics, microbial metabolic networks, and nitrogen removal efficiency elucidated in this study provides actionable strategies for advancing wastewater treatment systems. Overall, both stage-specific corn-starch wastewaters supported efficient nitrate removal under the tested conditions, while their contrasting compositions led to distinct nitrite-reduction behaviors and microbial responses. The structural complexity of carbon sources dictates microbial metabolic pathways and community dynamics. The molecular weights (MW) distribution of DOM in VSW and HAW ranged from 0.2 to 0.5 kDa, accounting for 57.02% and 57.06% of the total molecules, respectively. These lower MW compounds (<0.5 kDa) are predominantly aliphatic and organic acids, indicating a higher bioavailability.

Nevertheless, the distinct nitrogen removal efficiencies observed between HAW and VSW systems stem from their contrasting organic compositions and bioavailability. The VSW comprised by branched amylopectin and cellulose undergoes hydrolysis to generate diverse intermediates such as oligosaccharides and organic acids.^29^ These substrates sustain a synergistic cross-feeding microbial network. Specifically, Firmicutes (14.76% relative abundance) hydrolyze complex polymers into fermentable compounds, which subsequently fuel *Rhizobium* (52.4% relative abundance) and enhance *nirK*-mediated denitrification (76.61% relative abundance). This syntrophic interaction minimizes intermediate accumulation, achieving a denitrification rate comparable to that of SA. Previous evidence indicates that acidogenic liquid can exhibit slightly higher carbon utilization efficiency than SA, attributable to its rich content of diverse VFAs and trace elements.^53^ This is attributed to the closer alignment between the carbon source composition and microbial metabolic requirements, coupled with high bioavailability. In contrast, HAW, dominated by VFAs and amino acids derived from anaerobic acidification, preferentially enriched *nirS*-type denitrifiers (*Thauera* and *Rubrivivax*). Although VFAs are bioavailable, their linear metabolic pathways limit cross-feeding interactions, leading to electron competition between nitrate reductase (*narG*) and nitrite reductase (*nirS*), as evidenced by a slower nitrite reduction rate. This observation aligns with established studies indicating that branched carbon structures support syntrophic networks, whereas simple organic compounds restrict metabolic versatility.^54^ Therefore, carbon sources should be strategically tailored to balance structural complexity and metabolic versatility, which is a critical guideline for selecting natural bio-based carbon sources in future wastewater treatment applications. The underlying mechanisms warrant further investigation in subsequent work.

Community-level patterns further reinforce these implications. The consistent dominance of Proteobacteria (63.90%–65.29%) and Firmicutes (14.76%–14.86%) across systems highlights their complementary roles. Proteobacteria (e.g., *Thauera* and *Rubrivivax*) primarily drive *nirS*-mediated denitrification but often require aromatic or cyclic substrates. Conversely, Firmicutes specialize in hydrolyzing recalcitrant polymers into bioavailable intermediates. Notably, *Rhizobium* biomass was 10-fold higher in VSW (52.4%) compared to HAW (5.1%), correlating with its metabolic capacity to utilize acetate via the TCA cycle, thereby generating NADH for *nirK*-catalyzed nitrite reduction.^32^ Conversely, the glucose-dominated metabolism in HAW enriches *Thauera* (6.57% relative abundance), which employs *narG*/*narI* genes for nitrate reduction but contributes to nitrite accumulation.^55^ Literature suggested that denitrification enzyme activity is closely linked to the community structure of the *nirK*-type denitrifier community, but not to that of *nirS*-type denitrifier community or the absolute abundance of either functional group.^56^ In starch wastewater denitrification systems, *Rhizobium* was identified as the prominent type of *nirK-*type bacterium, demonstrating significant tolerance to NO and efficient nitrite reduction capability. The VSW system exhibited unique *nirK* denitrifiers, namely *Bosea*, *Sinorhizobium*, and *Paracoccus*, while the HAW system contained *Ochrobactrum* as the unique *nirK* denitrifier. This provided further evidence that *nirK* denitrifying bacteria species are more responsive to substrates. Inoculating systems with *Rhizobium* or *nirK*-enriched consortia could enhance nitrite reductase activity. Additionally, maintaining functional redundancy (e.g., *Nitrospirae*) ensures stability under fluctuating carbon loads. Together, these results suggest that VSW is particularly promising when the operational priority is to accelerate nitrite reduction and mitigate nitrite accumulation, whereas HAW may require tighter process control to achieve consistently complete denitrification.

Process monitoring implications are also apparent from pH dynamics. The pH trajectory reflected metabolic shifts during denitrification. During stage I, alkaline byproducts elevate pH (ΔpH = 1.1–1.5), with slower increases in HAW due to transient acidification by VFAs. In stage II, sharper pH rises correlate with OH^−^ generation, but HAW was limited organic acid pool restricts buffering capacity, exacerbating nitrite accumulation. At the end of denitrification, pH decline signals substrate exhaustion and acidogenic metabolism, offering a diagnostic marker for process termination. Real-time pH trajectories could complement online NOx monitoring to support more responsive carbon dosing, particularly when nitrite reduction becomes rate-limiting. From an operational standpoint, pH variation offers an informative window into the denitrification process. By monitoring pH profiles in real-time, operators can infer whether the system is in nitrate-reducing, nitrite-reducing, or fermentation-dominated stages. In advanced processes, such as partial denitrification or anaerobic ammonium oxidation, manipulating pH can help optimize NO_2_^−^ accumulation and tailor the system toward desired nitrogen removal pathways.^48^ Consequently, understanding and controlling pH not only helps track the progression of denitrification but also provides a potential strategy to balance acid/base production, improve alkalinity usage, and reduce external alkalinity demand.

From an engineering perspective, these kinetic and microbial differences translate into practical implications for process control and carbon-source selection. The 30-day monitoring supports the operational feasibility of reusing CSW as an external carbon source in SBR operation. Under the optimized operating conditions applied here (40 °C, influent C/*N* 7), nitrate removal in the anoxic stage reached about 97%, while the estimated operating cost of recycling CSW as the carbon source was CNY 0.08 m^-3^, dominated by energy consumption. Among the two stage-specific wastewaters, VSW appears more suitable when the operational priority is to accelerate nitrite reduction and minimize nitrite accumulation, consistent with its stronger *nirK*-type denitrifier response and higher denitrification potential with a lower sludge yield. In practice, VSW dosing could be regulated by a feedback control strategy (e.g., maintaining a target NO_3_^−^-N set-point in the anoxic zone), and integrating pH trajectories may further support phase identification and responsive carbon dosing, thereby improving stability and potentially mitigating N_2_O risks.

This study demonstrates the viability of CSW as a sustainable carbon source for efficient nitrogen removal, achieving over 97% nitrate elimination under optimized conditions (C/*N* = 7, 40 °C, pH 7). The composition of CSW significantly shaped microbial community dynamics. VSW, rich in starch-derived oligosaccharides, selectively enriched *Rhizobium* (52.4% abundance), a dominant *nirK*-type denitrifier critical for mitigating nitrite accumulation, while HAW favored *nirS*-type taxa (*Thauera*, *Rubrivivax*) linked to incomplete denitrification. Overall, this work supports a circular economy paradigm by recycling organic-rich effluents back into biological processes, potentially reducing chemical reagent usage. Future investigations should evaluate the long-term performance, comprehensive economic viability, and potential for simultaneous removal of other contaminants under real operating conditions.

### Limitations of the study

This study employed parallel lab-scale SBRs to enable a controlled comparison of stage-specific corn starch wastewaters (VSW and HAW) as external carbon sources. While this design allowed consistent kinetic and pH profiling under pseudo-steady-state conditions, the experiments were limited to representative operating conditions (e.g., influent C/N of 7°C and 40°C). Evaluations across broader temperature ranges, loading fluctuations, and continuous-flow configurations will further clarify robustness for full-scale implementation.

The inoculum sludge was collected from the same corn starch facility that generated VSW and HAW, which improves relevance for in-plant reuse scenarios; however, prior acclimation may lead to more optimistic absolute performance than would be observed with non-acclimated inocula. Future studies should therefore validate the findings using sludge from other sources (e.g., municipal WWTP biomass) to assess generalizability. Microbial insights in this work were primarily derived from 16S rRNA and nir-gene amplicon sequencing; mechanistic understanding would be strengthened by incorporating activity-based approaches (e.g., transcriptional profiling and enzyme assays) and investigating interspecific interactions within nirS- and nirK-harboring communities. Finally, a comprehensive techno-economic assessment will be valuable to evaluate implementation readiness of this circular strategy.

## Resource availability

### Lead contact

Requests for further information and resources should be directed to and will be fulfilled by the lead contact, Haihong Yan (yanhh@craes.org.cn).

### Materials availability

This study did not generate new unique reagents.

### Data and code availability

The raw 16S rRNA and *nirS/nirK* gene sequencing data generated during this study have been deposited at NCBI Sequence Read Archive (SRA) and are publicly available as of the date of publication. Accession numbers are listed in the key resources table. All other data generated or analyzed during this study are included in the manuscript and supplementary tables. Any additional information required to reanalyze the data reported in this paper is available from the lead contact upon request. This paper does not report original code.

## Acknowledgments

This work was financially supported by the Joint Research on Ecological Protection and High-Quality Development in the Yellow River Basin (2022-YRUC-01-0203).

## Author contributions

Investigation, H.Y. and X.G.; data curation, X.G. and Y.L.; software, Y.L. and F.B.; writing – original draft, X.G.; writing—review and editing, H.Y; funding acquisition, Y.N.

## Declaration of interests

The authors declare no competing interests.

## STAR★METHODS

### Key resources table

**Table:** 

REAGENT or RESOURCE	SOURCE	IDENTIFIER
**Antibodies**		

N/A	N/A	N/A

**Bacterial and virus strains**		

N/A	N/A	N/A

**Biological samples**		

Wastewater sludge	A corn deep-processing plant in Jilin Province, China.	N/A
Activated sludge samples collected from SBR reactors during operation	This paper	PRJNA1455738

**Chemicals, peptides, and recombinant proteins**		

Wastewater	A corn deep-processing plant in Jilin Province, China.	N/A
KH_2_PO_4_	Sigma-Aldrich	CAS: 7778-77-0
MgSO_4_·7H_2_O	Sigma-Aldrich	CAS: 10034-99-8
CaCl_2_·2H_2_O	Sigma-Aldrich	CAS: 10035-04-8
KHCO_3_	Sigma-Aldrich	CAS: 298-14-6
trace elements, see Table S1	Wang et al.^[^^57^^]^	N/A

**Critical commercial assays**		

High throughput sequencing	Novogene(Beijing, China)	https://www.novogene.cn/

**Deposited data**		

Raw 16S rRNA and *nirS/nirK* amplicon sequencing data	This paper; NCBI SRA	BioProject: PRJNA1455738

**Experimental models: Cell lines**		

N/A	N/A	N/A

**Experimental models: Organisms/strains**		

N/A	N/A	N/A

**Oligonucleotides**		

16S rRNA V3-V4 primer 341F: CCTAYGGGRBGCASCAG	This paper	N/A
16S rRNA V3-V4 primer 806R: GGACTACNNGGGTATCTAAT	This paper	N/A
*nirS* primer cd3aF: GTSAACGTSAAGGARACSGG	This paper	N/A
*nirS* primer R3cdR: GASTTCGGRTGSGTCTTGA	This paper	N/A
*nirK* primer Copper583F: TCATGGTGCTGCCGCGYGANGG	This paper	N/A
*nirK* primer Copper909R: GAACTTGCCGGTKGCCCAGAC	This paper	N/A

**Recombinant DNA**		

N/A	N/A	N/A

**Software and algorithms**		

Origin 2021	OriginLab	https://www.originlab.com/
MATLAB R2017b	MathWorks	https://www.mathworks.com/
WPS office	KINGSOFT	https://www.wps.cn/

**Other**		

PB-20 pH meter	Sartorius	N/A
ICS-1000 Ion Chromatography	Dionex	N/A
DR1010 rapid COD photometric analyzer	HACH	N/A
F-7000 Fluorescence Spectrophotometer	Hitachi	N/A

### Experimental model and study participant details

This study does not use experimental methods typical in the life sciences.

### Method details

#### Corn starch wastewater treatment process

The corn starch production facility (located in Jilin, China) treats CSW using a combination of physical and biological processes. The wastewater treatment system comprises a sedimentation tank, a hydrolysis-acidification basin, an anaerobic basin, and an oxic basin in series with a treatment capacity of 3,500 m^3^ d^−1^. Figure S1 shows the treatment train.

In this study, two effluents were collected for use as external carbon sources. VSW was obtained from preliminary sedimentation; this step removed most suspended solids. The remaining supernatant still contained abundant biodegradable organic matter. HAW was collected from the hydrolysis-acidification stage, where macromolecular organics were partially broken down into small-molecule organic acids, making the wastewater more amenable to subsequent microbial utilization.

#### Wastewater characterization

Prior to the denitrification experiments, the main water quality parameters of the VSW and HAW samples were analyzed. VSW exhibited a COD_Cr_ of 12,234 ± 37 mg L^−1^, while HAW showed a slightly lower value of 10,871 ± 28 mg L^−1^. The NH_4_^+^-N concentration was relatively higher in HAW (44 ± 5 mg L^−1^) compared to VSW (24 ± 3 mg L^−1^), which may reflect partial hydrolysis of proteins in the acidification step. Both wastewaters were mildly acidic, with pH values of 4.0 ± 0.1 (VSW) and 3.9 ± 0.1 (HAW). The nitrate and nitrite levels remained moderate: 4.9 ± 0.8 mg L^−1^ and 2.3 ± 0.3 mg L^−1^ in VSW, and 3.8 ± 0.7 mg L^−1^ and 2.8 ± 0.2 mg L^−1^ in HAW, respectively.

In terms of dissolved organic matter (DOM), each wastewater contained a broad range of molecular weights (MW) from approximately 0.13–23.4 kDa. VSW had a weight-average molecular weight to number-average molecular weight ratio (Mw/Mn) of 1.62, slightly broader than that of HAW (1.49). This distribution indicates that both wastewaters contain a mix of low-molecular-weight compounds (e.g., organic acids) and high-molecular-weight compounds. Overall, the acidic pH and relatively high COD_Cr_ values indicate that VSW and HAW remain rich in biodegradable organics, making them suitable candidates for use as external carbon sources to enhance denitrification processes.

**Table:** 

Water quality and DOM distribution of wastewater for denitrification testing							
Wastewater	COD_Cr_ (mg L^−1^)	NH_4_^+^-N (mg L^−1^)	NO_3_^−^ -N (mg L^−1^)	NO_2_^−^-N (mg L^−1^)	pH	MW (kDa)	Mw/Mn
VSW	12234 ± 37	24 ± 3	4.9 ± 0.8	2.3 ± 0.3	4.0 ± 0.1	0.14–23.1	1.62
HAW	10871 ± 28	44 ± 5	3.8 ± 0.7	2.8 ± 0.2	3.9 ± 0.1	0.13–23.4	1.49

#### SBR setup and operation

Three identical 7 L sequencing batch reactors (SBRs) were operated in parallel and are hereafter denoted R-VSW, R-HAW, and R-SA. Each vessel was fitted with a 50 rpm paddle-type impeller to keep the biomass uniformly suspended while avoiding oxygen entrainment.

Activated sludge was taken from the secondary clarifier of the corn starch plant that supplied the wastewaters. The sludge was rinsed three times with ultrapure water, then aerated for 30 min to remove residual organics and bring the cells to an endogenous-respiration state. The conditioned biomass, with an initial mixed liquor suspended solids (MLSS) concentration of 5.0 g L^−1^, was divided equally among the three reactors.

KNO_3_ was supplemented to achieve an initial NO_3_^−^-N concentration of 100 mg L^−1^. Each carbon source (HAW, VSW, and SA) was then added separately to adjust the soluble chemical oxygen demand (SCOD) to 700 mg L^−1^, corresponding to a C/N ratio of 7.0 ± 0.2. Following the methods of Wang et al.,^57^ all feeds contained the same basal mineral medium, including KH_2_PO_4_ (27.2 mg L^−1^), MgSO_4_·7H_2_O (300 mg L^−1^), CaCl_2_·2H_2_O (180 mg L^−1^), KHCO_3_ (500 mg L^−1^), and 1 mL L^−1^ of trace elements A and B (Table S1).Water samples were periodically removed to monitor the changes in NO_3_^−^-N, NO_2_^−^-N and SCOD.

Each SBR followed a fixed 6 h cycle, including 0.5 h feed, 4.5 h anoxic reaction, 0.5 h settling and 0.5 h decanting. With an exchange ratio of 0.50 (3.5 L withdrawn per cycle) the hydraulic-retention time was 12 h. The temperature was held at 40 ± 0.5 °C (matching the plant’s 38°C–42°C range), and dissolved oxygen was maintained below 0.4 mg L^−1^. Four cycles were performed daily, giving 24 h continuous operation and constant biomass levels. Sludge wasting was implemented using a threshold-triggered approach to maintain biomass at a target MLSS of approximately 5.0 g L^−1^. MLSS was measured every 2 days. When MLSS exceeded threshold value, a portion of well-mixed liquor was withdrawn from the reactor to reduce MLSS back to 5.0 g L^−1^, while keeping the working volume constant by replacing the withdrawn volume with an equal volume of influent.

Reactor performance was monitored until nitrate and nitrite removal efficiencies fluctuated by less than 5% over three successive cycles, a criterion that marked pseudo-steady state. Steady state was typically reached after about 14 days (56 cycles) for VSW and HAW and 10 days (40 cycles) for SA.

##### Liquid-phase sampling

During three independent steady-state cycles, 10 mL aliquots were drawn from each reactor at 0, 5, 10, 15, 20, 25, 30, 35, 40, 45, 50, 55, 60, 70, 80, 90, 100, 110, 120, 140, 160, 180, 210, 240, 270min of the anoxic phase, immediately filtered through 0.45 μm cellulose-acetate membranes, and stored at 4 °C in the dark.

##### Sludge sampling

At the end of the same three cycles, 50 mL of mixed liquor was withdrawn from mid-depth, centrifuged (4,500 g, 10 min, 4 °C), and the pellet was flash-frozen at −80 °C. The three biological replicates were processed separately for DNA extraction and 16S-rRNA amplicon sequencing. All chemical assays were run on three biological replicates, each measured in duplicate to provide technical precision.

#### Analytic methods

The samples were analyzed to measure the variations in pH, NO_3_-N, NO_2_-N and SCOD. pH was measured using a Sartorius PB-20 (PB-S) pH meter. The concentrations of NO_3_-N and NO_2_-N were determined using ion chromatography on an ICS-1000 (Dionex, USA). The measurements of SCOD was made with reference to detection methods of COD photometric rapid detector (HJ 924–2017).

The EEM was measured using the HITACHI F-7000 fluorescence instrument. Excitation and emission wavelengths were scanned from 200 nm to 500 nm. Additionally, a slit width of 5 nm, a photomultiplier tube voltage of 700 V, and a scanning speed of 12,000 nm min^−1^ were employed. Furthermore, a reference spectrum was collected by analyzing the ultrapure water. Before PARAFAC modeling, raw EEMs were pre-processed as follows: (i) ultrapure-water blanks were subtracted from all spectra; (ii) first- and second-order Rayleigh and Raman scattering bands were masked; (iii) inner-filter effects were corrected using paired absorbance spectra; and (iv) fluorescence intensities were normalized to Raman units (RU) based on the integrated area of the water Raman peak. These pre-processing steps were applied to improve the transparency and reproducibility of the EEM-PARAFAC analysis.

#### High-throughput sequencing

To examine variations in microbial community structure in response to different carbon sources, sludge samples were collected from three treatment conditions: (i) HAW supplementation, (ii) VSW addition, and (iii) a control group without carbon source amendment, followed by comprehensive analysis using 16S rRNA gene sequencing. All microbial samples were stored at −20°C before testing. The genomic DNA was extracted using the CTAB method, and its purity and concentration were checked with agarose gel electrophoresis. The samples were diluted to 1 ng μL^−1^ using sterile water. For amplification, the V3-V4 region of the bacterial 16S rRNA gene was amplified with specific primers 341F (CCTAYGGGRBGCASCAG) and 806R (GGACTACNNGGGTATCTAAT). The primers for the nirS functional genes were cd3aF (GTSAACGTSAAGGARACSGG) and R3cdR (GASTTCGGRTGSGTCTTGA), while those for the nirK functional genes were Copper583F (TCATGGTGCTGCCGCGYGANGG) and Copper909R (GAACTTGCCGGTKGCCCAGAC). The PCR reaction procedure included pre-denaturation at 98°C for 1 min, followed by 10 s at 98°C, 30 s at 50°C, and 30 s at 72°C for a total of 30 cycles, concluding with a final extension at 72°C for 5 min. After mixing and purifying the PCR products, the amplicons were sequenced on an HiSeq2500.

#### Data analysis

Denitrification rates were calculated as follows.

(Equation 3) $$C_{{\text{NO}}_{x}-N}=C_{{\text{NO}}_{3}^{-}-N}+0.6C_{{\text{NO}}_{2}^{-}-N}$$

(Equation 4) $$\text{VNOx}-N=\Delta \left({\text{NO}}_{x}^{-}-N\right)/\text{MLVSS}\cdot \Delta t$$

(Equation 5) $$P_{DN}=\Delta \left({\text{NO}}_{x}^{-}-N\right)/\Delta \text{SCOD}$$

(Equation 6) $$Y_{DN}=1-2.86P_{DN}$$

Where *C* is the concentration of NO_x_-N, NO_3_^−^-N and NO_2_^−^-N (mg L^−1^); *V*_NOx-N_ is the denitrification rate (mg N g^−1^ MLVSS h^−1^); MLVSS represents the volatile suspended solids concentration (g L^−1^); t represents the endpoint of calculation (h). P_DN_ is the potential of denitrification; Y_DN_ is the yield of denitrification;ΔNO_x_-N, ΔSCOD is the difference in concentration of NO_x_-N, SCOD before and after denitrification.

PARAFAC modeling was performed in MATLAB R2017b using the DOM-Fluor toolbox. Split-half validation and visual inspection were used to identify the PARAFAC components. The relative abundance of each fluorescent component was subsequently expressed by its maximum fluorescence intensity (Fmax). Statistical analysis was carried out using Origin 2021. OTUs were defined at 97% sequence similarity.

### Quantification and statistical analysis

The presented data in this paper are in average value ±SD with triplication.

### Additional resources

This study did not generate additional resources.
