# Supplementary material for: Organic composition of starch wastewater steers denitrifying microbiomes for enhanced nitrogen removal
Source: iScience. 2026 Jun 2;29(6):116217. doi: 10.1016/j.isci.2026.116217 (PMC13254897; doi:10.1016/j.isci.2026.116217)
Supplement: Document S1. Figures S1 and S2, Tables S1–S3 [file mmc1.pdf]

iScience, Volume *29*

Supplemental information

Organic composition of starch wastewater steers denitrifying microbiomes for enhanced nitrogen removal

Xiaoya Guo, Yu Liang, Haihong Yan, Yuegang Nian, and Fengyun Bu

Fig. S1 Schematic diagram of the wastewater treatment system

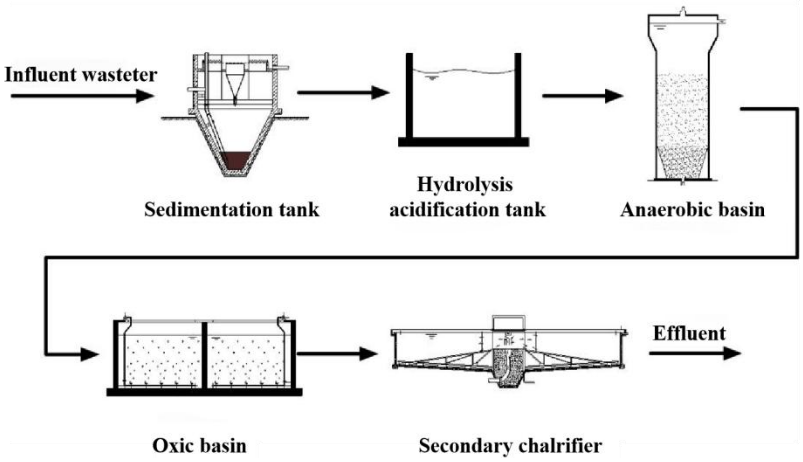

Fig.S2 Three fluorescence components identified by the PARAFAC model along with the excitation(solid curves) and emission (dotted curves) loading

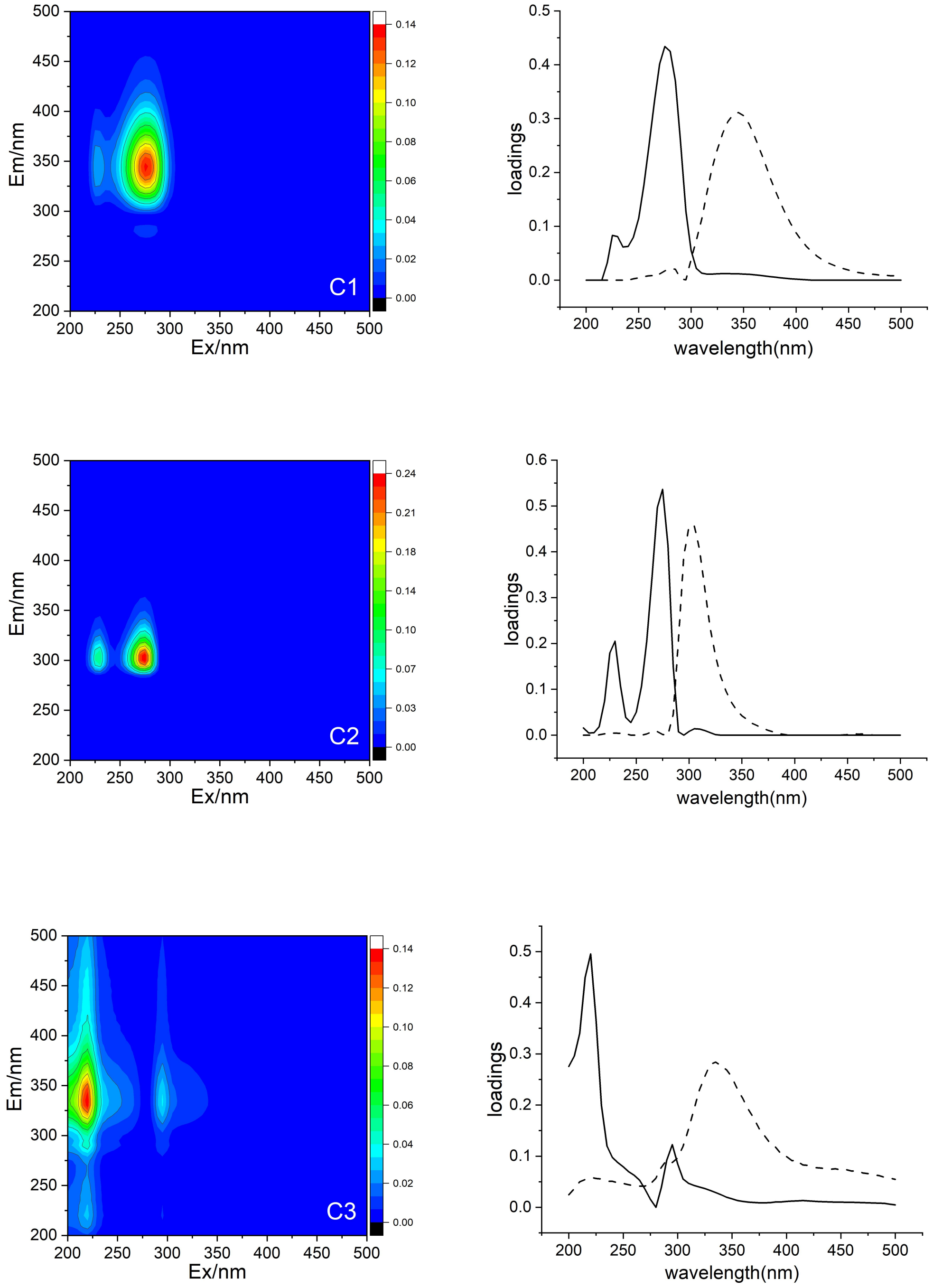

Table S1 Specific parameters of the reactor water distribution

Category	Components	Molecular mass	Masterbatch concentration	Dosage
Mineralogical composition	$MgSO_4 \cdot 7H_2O$	246	150 g/L	2 ml/L
	$CaCl_2 \cdot 2H_2O$	146.98	90 g/L	2 ml/L
	KH_2PO_4	136.09	13.6 g/L	2 ml/L
trace elements A	EDTA·2Na	338.22	6.369	1 ml/L
	$FeSO_4 \cdot 7H_2O$	278.05	9.14	
trace elements B	EDTA·2Na	338.22	19.11	1 ml/L
	H_3BO_4	61.83	0.014	
	$ZnSO_4 \cdot 7H_2O$	161	0.43	
	$CoCl_2 \cdot 6H_2O$	237.93	0.24	
	$MnCl_2 \cdot 4H_2O$	161.87	0.99	
	$CaSO_4 \cdot 5H_2O$	249.68	0.25	
	$NiCl_2 \cdot 6H_2O$	237.6	0.19	
	$NaMnO_4 \cdot 2H_2O$	241.92	0.22	

Table S2 Pearson correlation coefficients between fluorescent components and nitrogen species during denitrification in the HAW system (n = 23)

Phase	Component	NO_3^-	NO_2^-	NOx	C1	C2	C3
Stage I	NO_3^-	1	-0.997**	0.999**	-0.228	0.903**	-0.901**
	NO_2^-	-0.997**	1	-0.993**	0.259	-0.888**	0.890**
	NOx	0.999**	-0.993**	1	-0.210	0.909**	-0.905**
	C1	-0.228	0.259	-0.210	1	0.202	-0.190
	C2	0.903**	-0.888**	0.909**	0.202	1	-0.984**
	C3	-0.901**	0.890**	-0.905**	-0.190	-0.984**	1
Stage II	NO_3^-	1	0.564	0.565	0.573	0.225	-0.495
	NO_2^-	0.564	1	1.000**	0.993**	0.832**	-0.990**
	NOx	0.565	1.000**	1	0.993**	0.832**	-0.990**
	C1	0.573	0.993**	0.993**	1	0.846**	-0.976**
	C2	0.225	0.832**	0.832**	0.846**	1	-0.862**
	C3	-0.495	-0.990**	-0.990**	-0.976**	-0.862**	1

* $p < 0.05$; ** $p < 0.01$.

Table S3 Pearson correlation coefficients between fluorescent components and nitrogen species during denitrification in the VSW system (n = 23)

Phase	Component	NO_3^-	NO_2^-	NOx	C1	C2
	NO_3^-	1	-0.996**	0.999**	-0.861**	-0.872**
	NO_2^-	-0.996**	1	-0.990**	0.846**	0.886**
Stage I	NOx	0.999**	-0.990**	1	-0.866**	-0.859**
	C1	-0.861**	0.846**	-0.866**	1	0.875**
	C2	-0.872**	0.886**	-0.859**	0.875**	1
	NO_3^-	1	0.900**	0.900**	0.863*	-0.874*
	NO_2^-	0.900**	1	1.000**	0.914**	-0.953**
Stage II	NOx	0.900**	1.000**	1	0.914**	-0.953**
	C1	0.863*	0.914**	0.914**	1	-0.991**
	C2	-0.874*	-0.953**	-0.953**	-0.991**	1

* $p < 0.05$; ** $p < 0.01$.
